# CXCL10-LACTC1/C2 Expressing Mesenchymal Stem Cell Conditioned Medium Attenuates TNF-α-Induced Gene Expressions and Cell Viability in HUVECs

**DOI:** 10.1007/s10753-026-02518-2

**Published:** 2026-05-22

**Authors:** Kaya Molo, Ruken Ege, Kübra Ati̇k, Elif Çıtak, Orçun Çulcuoğlu, Hakan Darıcı, Emel Ordu

**Affiliations:** 1 Faculty of Science and Letters, Department of Molecular Biology and Genetics, Yıldız Technical University, Istanbul, 34220 Turkey; 2 Tissuecare Biotechnology, Istanbul, 34010 Turkey; 3 Faculty of Medicine, Department of Histology and Embryology, Istinye University, Istanbul, 34010 Turkey; 4 Stem Cell and Tissue Engineering R&D Center, Istinye University, Istanbul, 34010 Turkey

**Keywords:** CXCL10, Inflammation, Immunomodulation, Genetically Modified MSCs, Conditioned Medium

## Abstract

Infectious diseases exacerbate atherosclerosis-associated morbidity and mortality by inducing sustained inflammatory responses characterized by elevated IL-6, TNF-α, IFN-γ, and CXCL10. Persistent CXCL10-driven recruitment of CXCR3⁺ immune cells promotes endothelial dysfunction and atherosclerotic progression. Although mesenchymal stem cells (MSCs) respond to inflammatory cues and secrete CXCL10, the contribution of CXCL10/CXCR3 signaling to intrinsic MSC immunomodulatory programming remains poorly understood. The objective of this study is to investigate whether CXCL10 signaling can modulate MSC-mediated immunoregulation. To address this, Wharton’s jelly–derived MSCs (WJ-MSCs) were genetically engineered to express a membrane-anchored CXCL10–Lactadherin C1/C2 fusion protein (CXCL10-LACTC1/C2). This strategy was designed to recapitulate physiological, localized, and sustained CXCL10 signaling, enabling spatially restricted chemokine presentation that more closely mimics cell-associated CXCL10 in inflammatory microenvironments compared with soluble CXCL10. The Lactadherin C1/C2 domain was selected to achieve stable, physiological membrane anchoring without introducing artificial transmembrane domains or compromising CXCL10 bioactivity. CXCL10-LACTC1/C2–expressing MSCs exhibited increased expression of key immunoregulatory mediators, including IDO1, TGF-β1, and IL4I1. Furthermore, conditioned medium derived from these MSCs attenuated TNF-α–induced inflammatory responses in human umbilical vein endothelial cells (HUVECs), as indicated by the modulation of endothelial activation and homeostasis markers (ICAM-1, PECAM-1, KDR, vWF, and NRF2) and improvement of cell viability. Collectively, these findings provide mechanistic insight into CXCL10-mediated MSC immunoregulation and support further investigation of MSC-based and cell-free therapeutic strategies aimed at mitigating CXCL10-driven endothelial inflammation in infection-associated vascular injury and atherosclerotic disease progression.

**Supplementary Information:**

The online version contains supplementary material available at 10.1007/s10753-026-02518-2.

## Introduction

Cardiovascular diseases (CVDs) remain among the leading causes of global morbidity and mortality, with atherosclerosis representing the principal pathological basis underlying ischemic heart disease and ischemic stroke [13]. Beyond classical metabolic and genetic risk factors, inflammation driven by infectious agents has been extensively implicated in the development and progression of atherosclerotic disease [2].

A broad range of viral and bacterial infections—including severe acute respiratory syndrome coronavirus 2 (SARS-CoV-2), *Chlamydia pneumoniae*, *Helicobacter pylori*, influenza viruses, hepatitis viruses, herpes simplex virus, and human immunodeficiency virus—have been associated with accelerated atherosclerotic pathology. These infections induce sustained production of pro-inflammatory cytokines and chemokines such as interleukin-6 (IL-6), tumor necrosis factor-α (TNF-α), interferon-γ (IFN-γ), and C-X-C motif chemokine ligand 10 (CXCL10). Depending on the intensity and duration of the inflammatory stimulus, this response may manifest as acute cytokine storm or persist as chronic low-grade inflammation, both of which profoundly affect vascular homeostasis [2, 3, 5].

Endothelial cells play a central role in sensing and integrating inflammatory signals during infection. Recognition of pathogen- and damage-associated molecular patterns (PAMPs and DAMPs), together with exposure to inflammatory cytokines such as IL-1, TNF-α, and IFN-γ, drives endothelial activation characterized by increased expression of adhesion molecules, dysregulated nitric oxide signaling, and enhanced leukocyte recruitment. When inflammatory signaling is prolonged or inadequately resolved, endothelial dysfunction may progress to apoptosis and loss of vascular integrity, thereby facilitating immune cell infiltration and promoting atherosclerotic plaque development and progression [6]

Mesenchymal stem cells (MSCs) have attracted considerable interest in immunology and regenerative medicine due to their capacity to modulate both innate and adaptive immune responses. Through the secretion of soluble mediators and extracellular vesicles, as well as through direct cell–cell interactions, MSCs regulate immune cell activation, differentiation, and trafficking. Among the chemokines implicated in MSC biology, CXCL10 has been extensively studied for its role in immune cell recruitment via CXCR3 and for its involvement in MSC migration and homing toward inflammatory microenvironments [4, 9, 10]. However, despite the well-established role of the CXCL10/CXCR3 axis in immune regulation, its direct contribution to shaping the intrinsic immunomodulatory phenotype of MSCs remains incompletely defined and has been explored only in a limited number of mechanistic studies.

In the present study, we aimed to investigate whether CXCL10-associated signaling can directly influence mesenchymal stem cell–mediated immunomodulation. To address this question, CXCL10 was anchored to the MSC membrane via fusion with the Lactadherin C1/C2 domain, enabling sustained and localized CXCL10 presentation. Using this approach, we examined the impact of membrane-associated CXCL10 on the expression of key immunoregulatory mediators in MSCs and evaluated whether conditioned medium derived from CXCL10-modified MSCs modulates TNF-α–induced inflammatory responses in human umbilical vein endothelial cells (HUVECs). Through this experimental framework, the study seeks to further elucidate the potential role of the CXCL10/CXCR3 axis in MSC-mediated immunoregulation within an inflammatory endothelial context.

## Material & Methods

### Culturing and Characterization of Human Wharton’s Jelly Mesenchymal Stem Cells (hWJ-MSCs)

Cryopreserved human Wharton’s jelly mesenchymal stem cells (hWJ-MSCs) (Tissuecare Biotechnology) were thawed in a 37 °C water bath. The thawed hWJ-MSCs were transferred to a centrifuge tube containing DMEM/F12 (ThermoFisher) and centrifuged at 200 ×*g* for 10 min. The supernatant was discarded, and the cell pellet was resuspended in 1 mL of DPBS (ThermoFisher). Viable cell count was determined by mixing the cell suspension with 0.4% trypan blue solution at a 1:1 ratio and using the Countess 3 Automated Cell Counter (ThermoFisher).

The growth medium for hWJ-MSCs consisted of DMEM/F12 (ThermoFisher) supplemented with 10% fetal bovine serum (FBS) (ThermoFisher), 4 mM L-glutamine (ThermoFisher), and 1% penicillin-streptomycin (ThermoFisher). Cells were cultured at 37 °C in a 5% CO₂ incubator (PHcbi, multigas incubator). hWJ-MSCs were seeded in culture flasks at a density of 5,000 cells/cm² and were passaged every three days when 75% confluence was reached. Cells were passaged using 0.05% Trypsin-EDTA (ThermoFisher). Cells at passage 3 were used for experiments.

To confirm the identity of hWJ-MSCs, flow cytometry was performed to analyze the expression of mesenchymal surface markers (CD105, CD90, and CD73) and the absence of hematopoietic/endothelial markers (CD34, CD45, CD11b, CD79a, and HLA-DR) according to ISCT criteria. Cells were gated based on forward scatter (FSC) and side scatter (SSC) parameters to exclude debris. Marker positivity was defined using unstained and isotype-matched control samples. The antibodies used included CD90-APC Mouse IgG2A, CD73-CFS Mouse IgG2B, CD105-PerCP Mouse IgG1 for positive markers (R&D Systems), and CD45-PE Mouse IgG1, CD11b-PE Mouse IgG2B, CD79A-PE Mouse IgG1, and HLA-DR-PE Mouse IgG1 for negative markers (R&D Systems). Aliquots containing 1 × 10^5^ cells in a 100 µL volume were transferred into 5 mL flow cytometry tubes. Cells were then labeled with 10 µL of each target-specific primary antibody or a Negative Marker Cocktail. To account for non-specific background fluorescence, corresponding isotype control antibodies and a Negative Isotype Cocktail (10 µL each) were used in parallel control groups. The cell-antibody mixtures were incubated for 30–45 min at room temperature in a light-protected environment. Post-incubation, the cells were washed with 2 mL of Staining Buffer to remove unbound antibodies. The final cell pellets were resuspended in 200–400 µL of Staining Buffer. Data acquisition was performed immediately using a flow cytometer (BD FACSCalibur).

## Reprogramming hWJ-MSCs to Express the CXCL10-LACTC1/C2 Chimeric Fusion Membrane Protein

The CXCL10-LACTC1/C2 chimeric fusion membrane protein was designed by fusing the amino acid sequence of human CXCL10 (IP10) protein (NP_001556.2) with the membrane domain of the human MFG-E8/Lactadherin protein (NP_005919.2), covering amino acids 72 to 386, without the use of a linker. The 1260 bp CXCL10-LACTC1/C2 DNA sequence was cloned into the pLENTI-III-EF1a-MFGE8-Vector (Applied Biological Materials) and was obtained in lyophilized form. The target gene is driven by the EF1a promoter, and puromycin, under the control of the SV40 promoter, was selected as the resistance gene.

For pseudovirus production, HEK 293TN cells (System Biosciences) were seeded at a density of 3 × 10⁶ cells in 100 mm petri dishes with 10 mL of culture medium containing 10% FBS, 4 mM L-glutamine, and DMEM (ThermoFisher) (Day 1). On Day 2, the cells reached 50–70% confluency, and the medium was refreshed. A 1.5 mL microcentrifuge tube containing 0.8 mL serum-free DMEM was prepared by adding 10 µg of a second-generation packaging plasmid mix (Applied Biological Materials) and 10 µg of the pLENTI-III-EF1a-MFGE8-Vector plasmid. Then, 40 µL of PureFection™ transfection reagent (System Biosciences) was added, vortexed, and incubated at room temperature for 15 min. The mixture was added dropwise onto the cell cultures and incubated at 37 °C with 5% CO₂. On Day 3, the medium was changed again. On Days 4 and 5, the supernatant containing the pseudoviruses was collected and centrifuged at 3000 x*g* for 15 min. The supernatant was transferred to a clean tube, and PEG-it™ Virus Precipitation Solution (System Biosciences) was added and mixed gently 5–6 times by hand. The mixture was incubated at 4 °C for 24 h. The pseudoviruses were then pelleted by centrifugation at 1500 x *g* for 30 min and resuspended in 200 µL of cold DPBS (ThermoFisher). The pseudoviruses were aliquoted and stored at −86 °C until use.

For transduction, hWJ-MSCs were seeded into 24-well culture plates at a density of 14,000 cells/cm² in growth medium. After 24 h, when the cells reached 50–75% confluency, the medium was replaced with a new medium containing 400 µL of DMEM/F12 (with 10% FBS and 4 mM L-glutamine), 2.5 µL TransDux™ virus transduction reagent (System Biosciences) and 100 µL Max Enhancer™ reagent (System Biosciences). The pseudovirus suspension was added to the wells at volumes ranging from 50 µL to 200 µL Supplementery Material 1. Cells were incubated for 72 h with the pseudoviruses at 37 °C in a 5% CO₂ incubator. Following transduction, cells underwent antibiotic selection using puromycin for 7 days.

## Collection of Conditioned Medium from CXCL10-LACTC1/C2 Expressing hWJ-MSC Cultures

CXCL10-LACTC1/C2^+^ hWJ-MSCs were cultured in growth medium until reaching 90–95% confluence. Then, the existing medium was replaced with serum-free medium and incubated for 48 h. After incubation, the cell supernatant was collected and centrifuged at 300 x *g* for 15 min. The supernatant was stored at −86 °C until use.

## Human Umbilical Vein Endothelial Cells (HUVECs) Cell Cultures

Cryopreserved human umbilical vein endothelial cells (HUVECs) (ThermoFisher) were thawed in a 37 °C water bath. The thawed HUVECs were transferred to centrifuge tubes containing Medium 200 (ThermoFisher) and centrifuged at 200 x *g* for 10 min. The supernatant was discarded, and the pellet was resuspended in 1 mL of DPBS. Viable cell count was determined by mixing the cell suspension 1:1 with 0.4% Trypan Blue solution and using the Countess 3™ automated cell counter system (ThermoFisher). The growth medium for HUVECs was Medium 200 supplemented with Large Vessel Endothelial Supplement (LVES) (ThermoFisher). HUVECs were seeded in culture flasks at a density of 5,000 cells/cm² in growth medium and expanded by renewing the medium every three days until approximately 75% confluence. Cells were then passaged using 0.05% Trypsin-EDTA.

HUVECs were seeded at a density of 5,000 cells/cm² in 24-well culture plates. Wells were divided into control and experimental groups. When cells reached 50% confluence, the medium was replaced with medium containing 10 ng/mL TNF-α (ThermoFisher) [1, 7, 8, 11]. Cells were incubated for 24 h. Subsequently, the medium was refreshed as follows: Group 1A received medium containing only 10 ng/mL TNF-α; Group 1B received medium containing 10 ng/mL TNF-α plus 30% (v:v) CXCL10-LACTC1/C2^+^ MSC-conditioned medium [12]; Group 1C received medium containing only 30% (v:v) CXCL10-LACTC1/C2 positive MSC-conditioned medium [12]. The control group medium was refreshed with only HUVEC growth medium. Cells were then incubated for 48 h. Each experiment was performed in triplicate. Changes in gene expression, cell viability, and proliferation according to the composition of the media were assessed in separate 24-well plates. HUVEC cultures, media used in these cultures, and total incubation times were given Supplementary Material 2.

## RNA Isolation and Polymerase Chain Reaction

Total RNA was isolated from control and experimental groups using a commercial total RNA isolation kit (Ecopure), according to the manufacturer’s instructions. Cells were lysed, mixed with ethanol, and applied to RNA binding columns. Following sequential washing steps, RNA was eluted in 50–100 µL elution buffer. RNA concentration and purity were assessed using a NanoDrop spectrophotometer, and samples were stored at −86 °C until further analysis.

Complementary DNA (cDNA) synthesis was performed using the High Capacity cDNA Reverse Transcription Kit (Applied Biosystems) in a Bio-Rad thermal cycler. For each reaction, 10 µL of 2× reverse transcription master mix was combined with 10 µL of RNA sample. The resulting cDNA products were quantified using a NanoDrop spectrophotometer prior to downstream applications.

Quantitative real-time PCR (qPCR) was conducted using PowerUp™ SYBR Green Master Mix (Applied Biosystems) on a StepOnePlus™ Real-Time PCR System. Each reaction was prepared in a final volume of 20 µL containing 10 µL of 2× SYBR Green Master Mix, 10 ng of cDNA template, 800 nM each of forward and reverse primer, and PCR-grade water. Thermal cycling conditions were optimized according to primer melting temperatures, followed by melt curve analysis to confirm amplification specificity. Primer sequences are provided in Supplementary Material 3.

Relative mRNA expression levels were calculated using the 2^−ΔΔCt^ method after normalization to housekeeping gene (*B2M*) (Supplementary Material 4) expression. Fold changes were determined by comparing TNF-α–stimulated (10 ng/mL) samples with non-stimulated controls.

### SDS-PAGE and Western Blotting

To collect proteins from CXCL10-LACTC1/C2 positive hWJ-MSCs, the cells were detached from their culture plates using 0.05% trypsin/EDTA treatment. They were then centrifuged at 200 ×*g*, and the pellet was lysed with RIPA lysis buffer containing protease and phosphatase inhibitors (RIPA Lysis Buffer System, Santa Cruz Biotechnology). The lysate was pipetted and incubated on ice for 10–15 min, followed by centrifugation at 14,000 ×*g* for 5 min. The supernatant containing the cellular protein lysate was collected and stored at −86 °C until use.

Protein concentration in the cell lysates was measured by the Bradford method (Quick Start Bradford Protein Assay, Biorad). Prior to analysis, the dye reagent containing 1× methanol and phosphoric acid was removed from 4 °C storage and allowed to warm to room temperature. The dye reagent tube was inverted several times to homogenize the contents. BSA standards at concentrations of 2, 1, 0.75, 0.5, 0.25, and 0.125 mg/mL were used as standards. A 0 mg/mL BSA standard (blank) was prepared using the dye reagent. In microplate wells, 5 µL of standards and samples were added in triplicate, along with 250 µL of dye reagent. After a 5-minute incubation at room temperature, absorbance was read at 595 nm using a microplate reader. The average measurements of the standards were graphed in Microsoft Excel, and protein concentrations were calculated based on the resulting linear regression equation.

SDS-PAGE gels consisted of two layers: a 12% resolving gel and a 4% stacking gel. To prepare the 10% SDS (Sigma-Aldrich) solution, a total of 10.0 g SDS was dissolved in 100 mL distilled water (dH_2_O). For the 10% APS (Sigma-Aldrich) solution, 0.1 g APS was dissolved in 1 mL dH_2_O. For the 12% resolving gel, 1.5 M Tris-HCl buffer (pH 8.8) was prepared by dissolving 18.15 g Tris base (Sigma-Aldrich) in 100 mL dH_2_O. For the 4% stacking gel, 0.5 M Tris-HCl buffer (pH 6.8) was prepared by dissolving 6.0 g Tris base in 100 mL dH_2_O. The 10X SDS running buffer, used to facilitate protein migration during electrophoresis, was prepared by dissolving 3.30 g Tris base, 144.0 g glycine (Sigma-Aldrich), and 10.0 g SDS in 1 L dH_2_O. For experiments, the 10X running buffer was diluted to 1X.

Samples containing at least 10 µg of protein were mixed 1:1 with 2X Laemmli buffer and incubated at 95–100 °C for 5 min. A 10–250 kDa prestained protein ladder (ThermoFisher) was loaded into the left and right wells of both gels. The gels were run at 200 V for 2–3 h. After electrophoresis, proteins were transferred onto 0.2 μm PVDF mini membranes (Biorad, Transfer Pack) using a semi-dry blotting system (Biorad, Trans Blot Turbo) in transfer buffer. The blotting protocol was set at 1 A constant current, 25 V, for 30 min.

Membranes were washed for 5 min with 1× TBST under agitation. Blocking was performed for 1 h in blocking buffer under agitation. After blocking, membranes were washed again for 5 min with 1× TBST. Then, membranes containing the cell lysates were incubated overnight at + 4 °C with CXCL10 primary antibody (ThermoFisher) diluted 1:3000 in blocking buffer under agitation. After incubation, membranes were washed once for 5 min with 1× TBST. Membranes were then incubated for 2 h with goat anti-rabbit IgG secondary antibody (AbCam) diluted 1:3000 in blocking buffer under agitation. Following incubation, membranes were washed three times for 5 min each with 1× TBST. Membranes were incubated for 5 min in the dark with HRP chemiluminescent substrate (Elabscience). Detection was performed using the iBright Imaging System (ThermoFisher).

## Immunofluorescence Analyses

hWJ-MSCs transduced with the CXCL10-LACTC1/C2 gene were labeled by immunofluorescence (IF) using an anti-CXCL10 antibody (ThermoFisher). For this, cells were seeded in 48-well culture plates and cultured for one day. The culture medium was then removed, and cells were fixed with 4% paraformaldehyde for 1 h. After washing three times with PBS, cells were blocked and permeabilized for 30 min in a solution containing 1% BSA (Sigma-Aldrich) and 0.3% Triton X-100 (Sigma-Aldrich). After removing the blocking solution, cells were stained with primary antibodies at the dilution ratios described in Supplementary Material 5 .

## MTT Analysis

Cell viability and proliferation rates were determined using the 3-(4,5-dimethylthiazol-2-yl)−2,5-diphenyltetrazolium bromide (MTT) assay. A stock solution of MTT (Sigma-Aldrich) was prepared at 5 mg/mL in phosphate-buffered saline (PBS) or Medium 200. The working solution was then prepared by diluting the stock to a final concentration of 0.5 mg/mL in HUVEC culture medium. After removing the existing medium from the 24-well plates, 200 µL of the MTT working solution was added to each well. Following a 3 h incubation at 37 °C in a 5% CO_2_ atmosphere, the medium was aspirated, and the resulting formazan crystals were dissolved in DMSO. Absorbance was measured at 570 nm, with 630 nm used as the reference wavelength, using a microplate reader. Cell viability was calculated as a percentage relative to the control group.

### Protein-Protein Interaction (PPI) Network Analysis

To elucidate the functional crosstalk between membrane-bound CXCL10 (fused with Lact-C1/C2 domain) and its receptor CXCR3 on MSCs, an in silico protein interaction model was constructed. The STRING database (version 12.0) was utilized to map the functional associations between the CXCL10-CXCR3 axis and key immunosuppressive effectors, including TGF-β1, IDO1, and IL4I1. The network was expanded to include master regulatory signaling nodes relevant to MSCs and regulatory T (Treg) cell biology, specifically NF-κB, AKT1, STAT1, STAT3, STAT6, MAPK1, MAPK3, and JAK2. Interactions were filtered based on a high-confidence score (≥ 0.700) and integrated through multiple evidence channels, including experimental assays, co-expression data, curated databases, and automated text mining. To identify discrete functional modules within the expanded network, the Markov Clustering (MCL) algorithm was applied with an inflation parameter of 3.0. This clustering approach allowed for the visualization of distinct signaling and effector sub-networks, providing a mechanistic basis for the CXCL10-mediated immunosuppressive phenotype.

### Statistical Analysis

Statistical analyses were performed using Microsoft Excel equipped with the Real Statistics Resource Pack. All experiments were conducted with a sample size of (*n* = 3) per group. Descriptive statistics are expressed as mean ± standard deviation (SD). Significant differences between groups were determined using one-way analysis of variance (ANOVA), followed by Tukey’s Honest Significant Difference (HSD) post hoc test. A p-value ≤ 0.05 was considered statistically significant.

All materials utilized in this study are provided in the Supplementary Material 6.

## Results

### Verification of Mesenchymal Stem Cell Characteristics

The proportion of cells carrying the CD45, CD11b, and CD34 antigens in the hWJ-MSC population was found to be 0.29%. In contrast, the proportion of CD90 antigen-positive cells in the cell population was 93.19% for CD90, 91.29% for CD105, 98.78% for CD44, and 99.48% for CD73 (Supplementar Material 7).

### Demonstration of CXCL10-LACTC1/C2 Chimeric Fusion Protein Expression in hWJ-MSCs

**Fig. 1:**
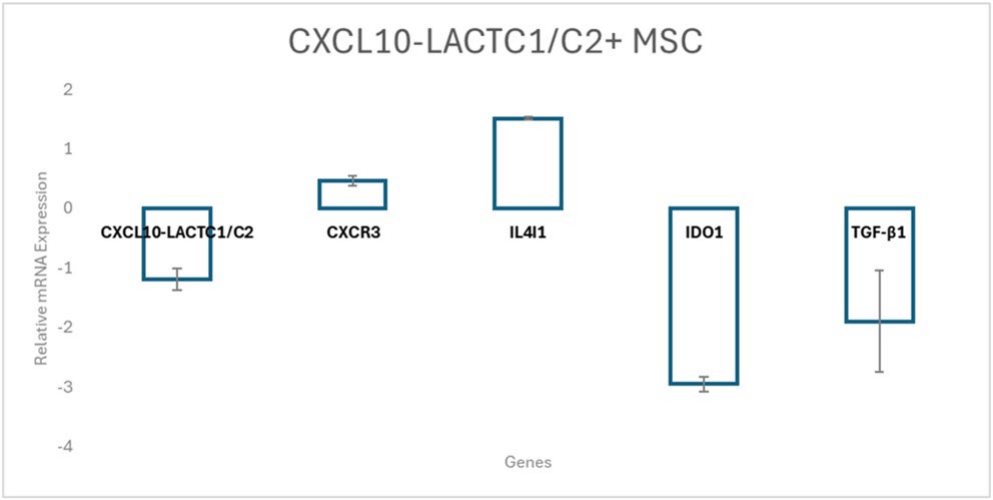
Log_2_2^(−∆Ct)^ Relative mRNA expression levels of genes (CXCL10-LactC1/C2, CXCR3, IL4I1, IDO1 and TGF-β1) for modified mesenchymal stem cells expressing CXC10-LactC1/C2

Quantitative real-time PCR (qRT-PCR) analysis revealed that the relative mRNA expression of CXCL10–LactC1/C2 in human Wharton’s jelly–derived mesenchymal stem cells (hWJ-MSCs), normalized to the *B2M* reference gene, was − 1.199 ± 0.183 on a log₂ scale. This corresponds to an expression level of approximately 43.5% relative to *B2M* (Fig. 1; Table 1).

**Fig. 2:**
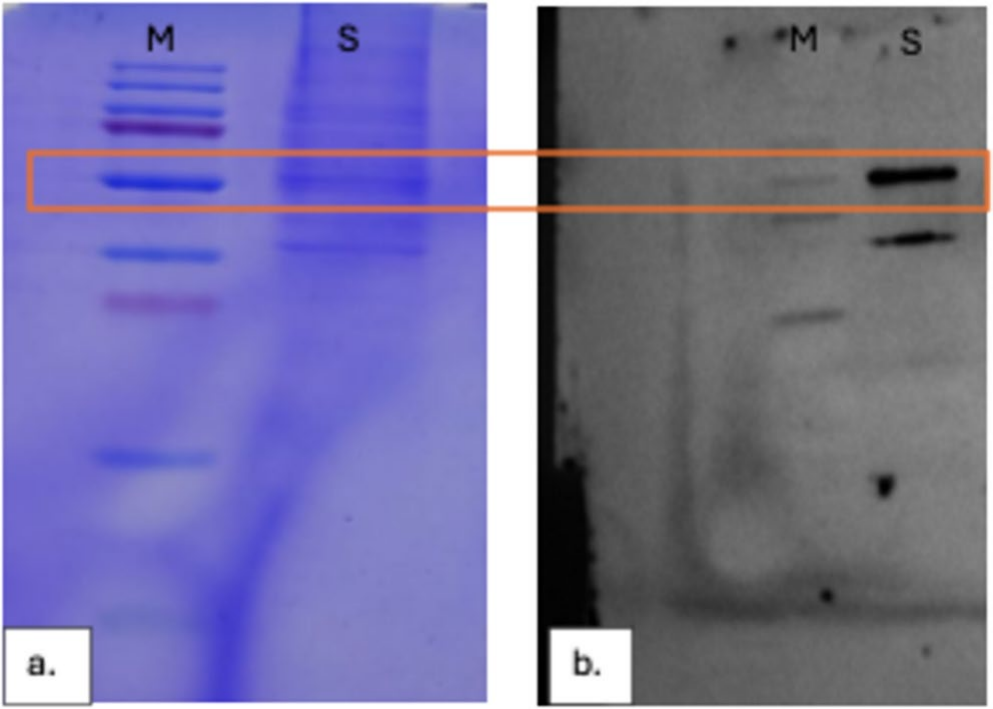
Detection of the CXCL10-LACTC1/C2 fusion protein via SDS-PAGE gel (**a**.) Western Blot analysis (**b**.). The presence of the protein was confirmed using a CXCL10-specific antibody, with an expected molecular weight of 48–55 kDa. The corresponding fusion protein band is indicated by the orange frame. M: Marker (PageRuler, 10–250 kDa prestained protein ladder (ThermoFisher)) and S: Sample

The protein concentration in lysates obtained from hWJ-MSCs expressing CXCL10-LACTC1/C2 was quantified as 0.958 mg/mL using the Bradford protein assay. Subsequent SDS-PAGE followed by Western blot analysis revealed the presence of the CXCL10-LACTC1/C2 fusion protein detected by a CXCL10-specific antibody (ThermoFisher) with an apparent molecular weight at approximately ranging between 48-55 kDa (Fig. 2). 

**Fig. 3:**
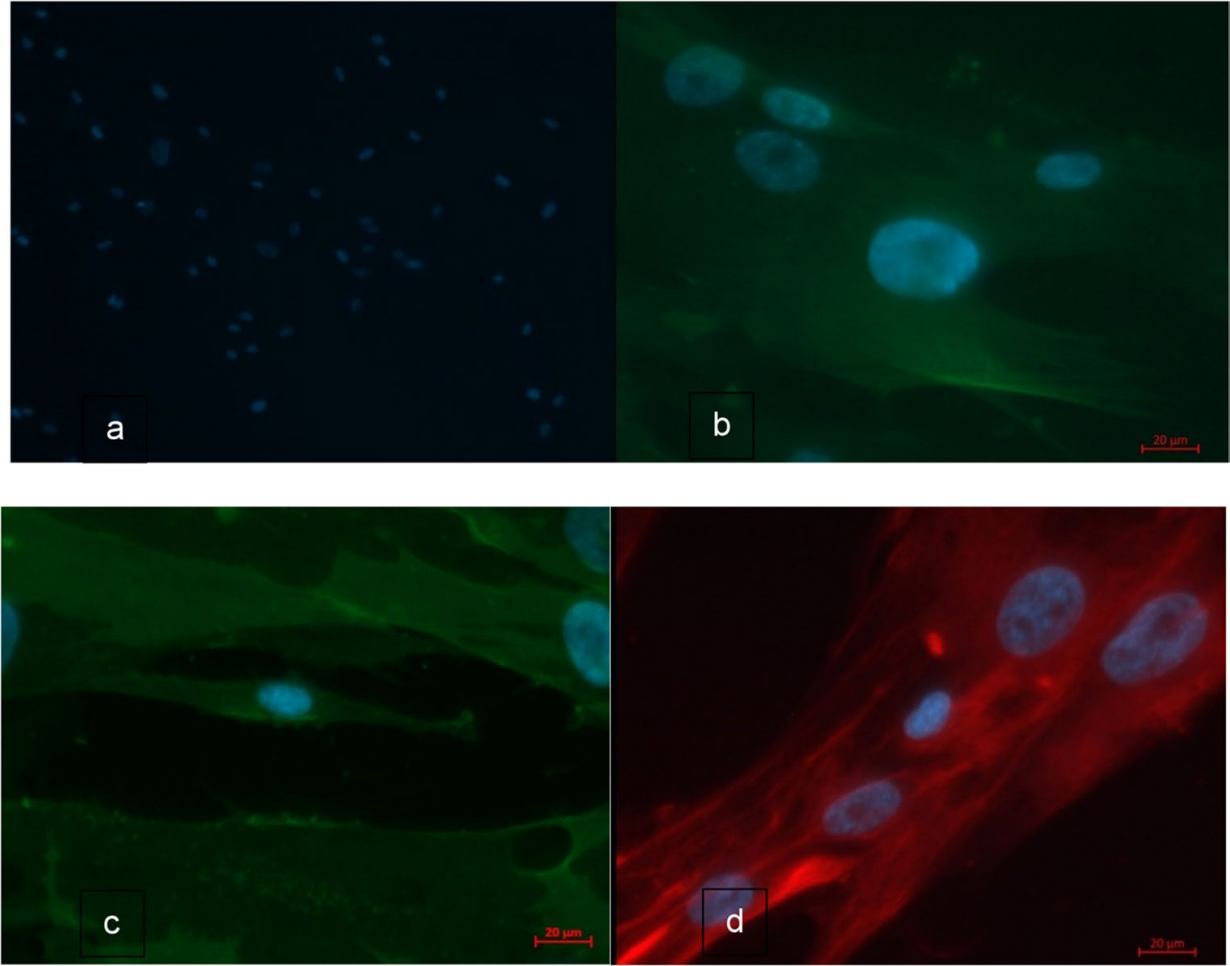
Immunofluorescence Microscopy Analysis: (**a**) Control MSCs lacking CXCL10/LACT-C1/C2 expression; (**b**) MSCs expressing CXCL10/LACT-C1/C2 (Green); (**c**) CD44 expression (Green) in MSCs expressing CXCL10/LACT-C1/C2; (**d**) Vimentin expression (Red) in MSCs expressing CXCL10/LACT-C1/C2. Cell nuclei were counterstained with DAPI(Blue)

In immunocytochemical staining experiments, the CXCL10 antibody (diluted 1:50) revealed membrane-associated staining in the cells, demonstrating specific membrane localization (Fig. 3). For the MSC marker CD44, fluorescence was concentrated at the membrane level with no nucleus-specific staining observed. No non-specific fluorescence was detected in the negative control wells where the primary antibody was omitted. Vimentin staining performed on the same samples also confirmed the expected MSC morphology (Fig. 3). The expressing of CXCL10-LACTC1C2 and mesenchymal stem cell markers (vimentin and CD44) were shown by immunofluorescence assay (IFA).

### Detection of Gene Expressions in hWJ-MSCs Expressing CXCL10-LACTC1/C2

The CXCL10-LACT-C1/C2 chimeric fusion protein, localized to the membrane surface of mesenchymal stem cells (MSCs), is hypothesized to exert its biological activity via interaction between its CXCL10 (IP-10) chemokine domain and the CXCR3 chemokine receptor present on the membrane surfaces of adjacent MSCs. To evaluate this potential mechanism, CXCR3 mRNA expression was assessed in MSCs expressing the CXCL10-LACT-C1/C2 chimeric fusion protein. Previous research has demonstrated that CXCL10 can induce M2 macrophage polarization under non-inflammatory conditions via LAMP-1 regulation [14]. Under non-inflammatory conditions, it was investigated whether the potential CXCL10–CXCR3 interaction in MSCs influences the expression of genes with anti-inflammatory functions, similar to its effects observed in macrophages. For this purpose, IL4I1, IDO1, and TGF-β1 mRNA expression levels were examined.

**Fig. 4:**
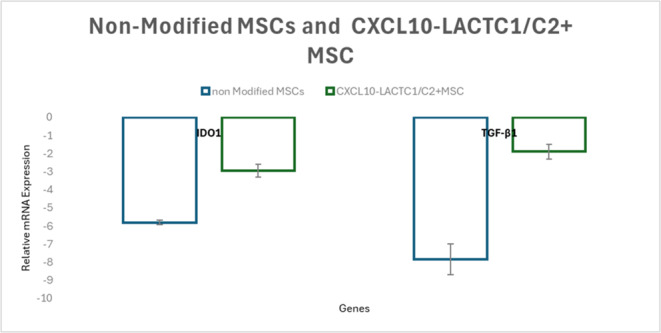
Log_2_2^(−ΔCt)^ Relative mRNA expression levels of genes (IDO1 and TGF-1β) for non-modified and modified mesenchymal stem cells expressing CXC10-LactC1/C2. Difference between expressions are statistically significant difference (p≤0.05)

Relative expressions of IDO1, and TGF-β1 were examined in unmodified WJ-MSC and CXCL10-LACTC1/C2^+^-WJ-MSC groups. Additionally, relative expressions of CXCR3 and IL4I1 were examined in CXCL10-LACTC1/C2^+^-WJ-MSC group. In the CXCL10-LACTC1/C2^+^ WJ-MSC cultures, the relative mRNA expression levels of CXCR3, IL4I1, IDO1, and TGF-β1 were 0.458 ± 0.079, 1.51 ± 0.02, −2.955 ± 0.125 and − 1.9 ± 0.85, respectively (Fig. 1; Table 1). In unmodified WJ-MSC cultures, the relative mRNA expression levels of IDO1 and TGF-β1 were − 5.81 ± 0.36 and 7.835 ± 0.405 (Fig. 4). The findings suggest that there is a statistically significant difference (*p* ≤ 0.05) between CXCL10-LACTC1/C2^+^ MSC and unmodified WJ-MSC cultures regarding IDO1 and TGF-β1 expression (Fig. 4; Table 2).

**Table 1:** Log_2_2^(−ΔCt)^ Relative mRNA Expressions and % Relative mRNA Expression Rates.

Genes	Log_2_2^(−ΔCt)^ Relative mRNA Expressions of CXCL10-LACTC1/C2^+^ MSC	% Relative mRNA Expression Rates of CXCL10-LACTC1/C2^+^ MSC
CXCL10-LACTC1/C2	−1.199 ± 0.183	43.5%
CXCR3	0,458 ± 0,079	137%
IL4I1	1,51 ± 0,02	285%
IDO1	−2,955 ± 0,125	13%
TGF-β1	−1,9 ± 0,85	27%

**Table 2:** Log_2_2^(−ΔCt)^ Relative mRNA Expressions and % Relative mRNA Expression Rates of MSCs and CXCL10-LACTC1/C2 + MSCs.

Genes	MSC		CXCL10-LACTC1/C2 + MSCs	
	Log_2_2^(−ΔCt)^ Relative mRNA Expressions	% Relative mRNA Expression Rates	Log_2_2^(−ΔCt)^ Relative mRNA Expressions	% Relative mRNA Expression Rates
IDO1	−5,81 ± 0,36	1,76%	−2,955 ± 0,125	13%
*N* = 3 Between Groups *p* = 0,017				
TGF-β1	−7,835 ± 0,405	0,44%	−1,9 ± 0,85	27%
*N* = 3 Between Groups *p* = 0,024				

### *In Silico* Validation of the CXCL10-Driven Immunosuppressive Regulatory Circuit

To delineate the intracellular signaling architecture bridging the CXCL10-CXCR3 axis with the primary immunosuppressive effectors IDO1, IL4I1, and TGF-β1, we constructed an expanded 14-node interactome. By integrating master regulatory hubs—including JAK2, STAT family members (1, 3, 6), NF-κB, and the MAPK/AKT signaling cascade—the network displayed a highly significant functional enrichment (PPI enrichment *p*-value: < 1.0e-16). The emergence of 43 observed edges, drastically surpassing the 8 expected edges, underscores a robustly integrated and non-random regulatory circuit. This high degree of connectivity suggests that membrane-tethered CXCL10 initiates a coordinated signaling program that transcriptionally stabilizes the potent enzymatic and cytokine profile of the programmed cells.

### Functional Modularization via MCL Clustering

**Fig. 5:**
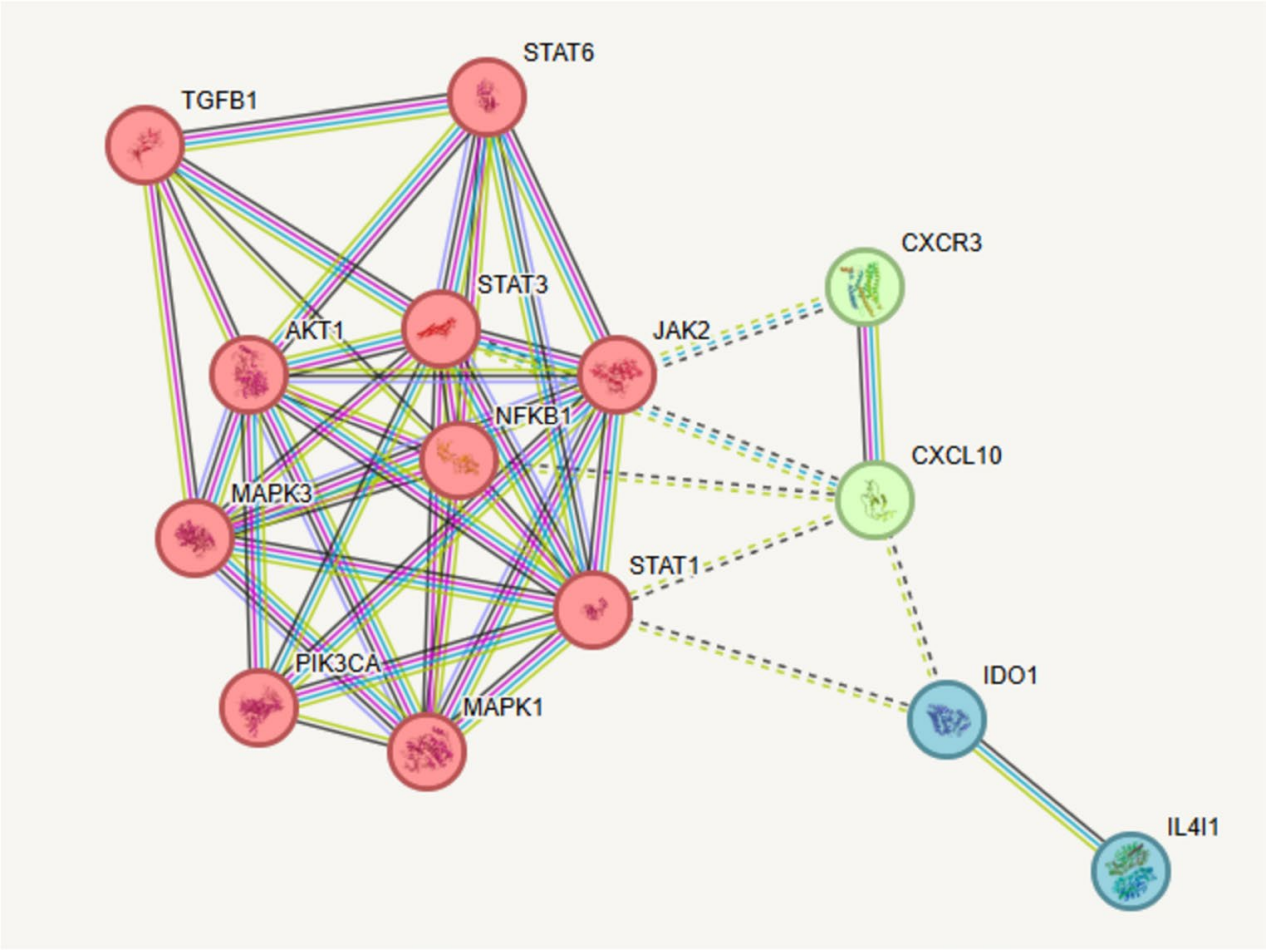
Protein-protein interaction (PPI) network of proteins involved in specific signaling pathways. While the red cluster (e.g., JAK2, STAT3, NFKB1) forms the core of the network, functional connections with proteins such as CXCL10 and IDO1 are indicated by dashed lines (PPI enrichment p-value:< 1.0e-16)

To further elucidate the topological organization of the 14-node interactome, the Markov Clustering (MCL) algorithm (with an inflation parameter: 3.0) was applied. This analysis partitioned the expanded network into three distinct functional modules, unveiling a clear hierarchical signaling flow that orchestrates the MSCs' immunosuppressive program. This hierarchy initiates with the Input Module (Green Cluster), centered on the CXCL10-CXCR3 axis, which serves as the primary trigger for autocrine and juxtacrine signaling loops. The spatial localization of membrane-tethered CXCL10 acts as a constitutive stimulus that is subsequently processed by the Signal Transduction Hub (Red Cluster), a dense regulatory core dominated by master transducers including JAK2, STAT1/3, and NF-κB. Notably, TGF-β1 occupies a central topological position within this hub; coupled with its extensive connectivity (average node degree: 6.14), this underscores the role of TGF-β1 as a key orchestrator that integrates multiple signaling inputs to stabilize the cellular phenotype. At the distal end of this cascade lies the Metabolic Effector Module (Blue Cluster), consisting of the potent immunosuppressive enzymes IDO1 and IL4I1, which were identified as direct downstream targets of the STAT and NF-κB signaling nodes. The transition from a decentralized interactome (Fig. 5) to this highly organized, modular architecture (Fig. 5) provides compelling in silico evidence for our hypothesis. Specifically, the data indicate that surface-localized CXCL10 expression triggers a coordinated JAK2/STAT-dependent signaling cascade that likely facilitates the transcriptional stabilization of *Ido1*, *Il4i1*, and *Tgfb1*, thereby reinforcing the potent immunosuppressive signature of the programmed MSCs.

### MTT Analysis

**Fig. 6:**
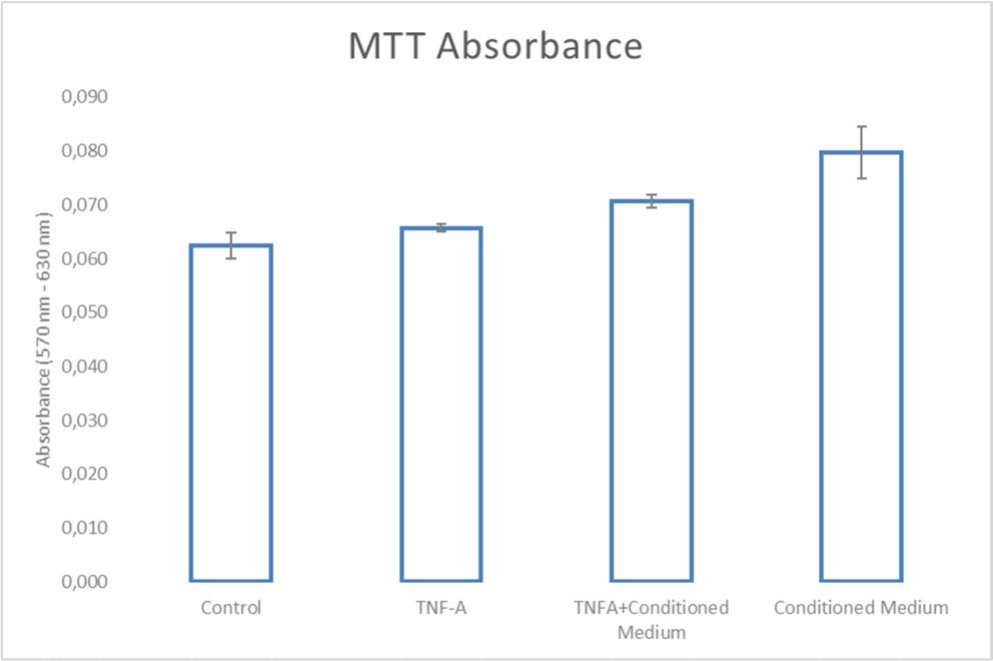
MTT assays showing the effect of CXCL10-LACTC1/C2 expressing MSC-conditioned medium on the viability of TNF-α-stimulated HUVECs. There is a statistically significant difference in MTT absorbance (N=3, Between Groups p = 0.002, Between TNF-A and TNF-A and TNF-A + Conditioned Medium Groups p=0,04; Between TNF-A and Conditioned Medium Groups p=0,04; )

**Fig.7:**
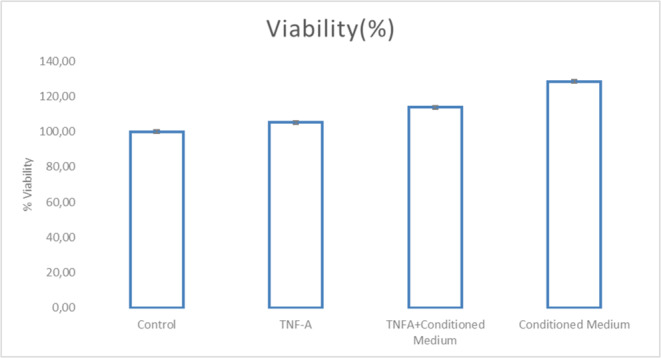
Cell viabilities according to average MTT absorbances

The MTT assay was performed to evaluate the effect of the conditioned medium obtained from CXCL10-LACTC1/C2^+^ MSCs on the viability of TNF-α-stimulated human umbilical vein endothelial cells (HUVECs). No significant difference in MTT absorbance was observed between the control HUVEC cultures and those preconditioned with 10 ng/mL TNF-α for 72 h. However, a significant increase in MTT absorbance (*p* = 0.03) was detected in HUVEC cultures that were preconditioned with 10 ng/mL TNF-α for 24 h, followed by 48 h of treatment with a medium containing 10 ng/mL TNF-α and 30% (v/v) CXCL10-LACTC1/C2^+^ MSC-conditioned medium, compared to cultures treated with TNF-α alone for 72 h. Furthermore, a statistically significant difference in MTT absorbance (*p* = 0.01) was observed between HUVEC cultures preconditioned with 10 ng/mL TNF-α for 72 h and those preconditioned with 10 ng/mL TNF-α for 24 h followed by 48 h of exposure to medium supplemented with 30% (v/v) CXCL10-LACTC1/C2^+^ MSC-conditioned medium (Figs. 6 and 7).

Accordingly, it was determined that 30% (v/v) conditioned medium derived from CXCL10-LACTC1/C2^+^ MSCs enhance the viability of TNF-α-stimulated HUVECs when applied following an initial inflammatory stimulus, indicating a potential protective or regenerative effect under inflammatory conditions (Fig. 7).

### Gene Expressions Analysis in HUVEC Cultures

The modulatory effects of CXCL10-LACTC1/C2⁺ MSC-derived conditioned medium on TNF-α–induced endothelial inflammation were assessed by analyzing the expression of key endothelial and redox-related genes in HUVEC cultures. Exposure to 10 ng/mL TNF-α significantly altered the expression of adhesion molecules and endothelial markers, whereas supplementation with 30% (v:v) CXCL10-LACTC1/C2⁺ MSC conditioned medium partially or completely reversed these effects.

**Fig. 8:**
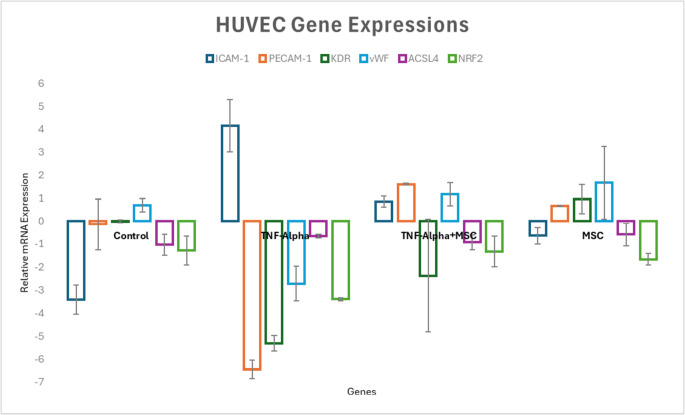
Quantitative analysis of the relative mRNA expression of genes in HUVECs. Induced HUVECS were treated by the conditioned medium with 10 ng/mL TNF-a or conditioned medium

TNF-α stimulation resulted in a marked upregulation of ICAM-1 expression (−3.4 ± 0,63 to 4.15 ± 1.14, *p* = 0.005), indicative of endothelial activation. This increase was attenuated in HUVECs cultured with TNF-α in the presence of CXCL10-LACTC1/C2⁺ MSC conditioned medium, which reduced ICAM-1 expression compared to TNF-α treatment alone (4.15 ± 1.14 to 0.84 ± 0.23, *p* = 0.03). Similarly, conditioned medium alone significantly suppressed ICAM-1 expression relative to TNF-α–treated cultures (4.15 ± 0.93 to −0.58 ± 0.35, *p* = 0.02) (Table 3; Fig. 8).

Conversely, TNF-α exposure significantly downregulated PECAM-1 (−0.4 ± 1.1 to −6.45 ± 0.33, *p* = 0,005) and KDR expression (−0.015 ± 0.065 to −5.3 ± 0.34, *p* = 0.005),reflecting impaired endothelial integrity and angiogenic potential. Co-treatment with CXCL10-LACTC1/C2⁺ MSC conditioned medium significantly restored the expression of PECAM-1 (−6.45 ± 0.33 to −1.6 ± 3.23, *p* = 0.002) compared to TNF-α–only conditions (Table 3; Fig. 8). Conditioned medium alone further enhanced the expression of PECAM-1 (−6.45 ± 0.33 to 0.67 ± 0.03, *p* = 0.003) and KDR (−3.995 ± 0.02 to 0.95 ± 0.64, *p* = 0.001).

TNF-α treatment also reduced vWF expression (0.69 ± 0.28 to −2.71 ± 0.75, *p* = 0.01). Although co-treatment with conditioned medium led to a partial increase in vWF expression, statistical significance was observed only in cultures treated with conditioned medium alone (−1.4 ± 0.75 to 1.,9 ± 0.07, *p* = 0.018) (Table 3; Fig. 8). In contrast, ACSL4 expression remained unchanged across all experimental conditions (Table 3; Fig. 8).

Analysis of redox-related signaling revealed that TNF-α significantly suppressed NRF2 expression (−1.27 ± 0.62 to −3.39 ± 0.07, *p* = 0.04) (Table 3; Fig. 8). This effect was significantly reversed by CXCL10-LACTC1/C2⁺ MSC conditioned medium under both co-treatment (−3.39 ± 0.07 to −1.32 ± 0.65, *p* = 0.04) and conditioned medium–only conditions (−3.39 ± 0.07 to −1.65 ± 0.26, *p* = 0.04) (Table 3; Fig. 8).

Collectively, these findings demonstrate that CXCL10-LACTC1/C2⁺ MSC-derived conditioned medium mitigates TNF-α–induced endothelial activation while preserving endothelial identity and redox homeostasis in HUVEC cultures.

**Table 3:** In Control and Assay Groups, Log_2_2^(−ΔCt)^ Relative mRNA Expressions and % Relative mRNA Expression Rates of HUVECs

Genes	Control Group	10 ng/mL TNF-α Assay Group	10 ng/mL TNF-α + Conditioned Medium Assay Group	Conditioned Medium Assay Group
ICAM-1	−3,4 ± 0,63 (9,47%)	4,15 ± 1,14 (1775,31%)	0,84 ± 0,235 (179%)	−0,64 ± 0,35 (64,17%)
*N* = 3 Between Groups *p* = 0,006 (One Way ANOVA) Between Control and 10 ng/mL TNF-α Assay Groups *p* = 0,003 (Tukey’s HDS) Between 10 ng/mL TNF-α Assay and 10 ng/mL TNF-α + Conditioned Medium Assay Groups *p* = 0,03 (Tukey’s HDS) Between 10 ng/mL TNF-α Assay Group and Conditioned Medium Assay Groups *p* = 0,01 (Tukey’s HDS)				
PECAM-1	−0,14 ± 1,1 (90,75%)	−6,45 ± 0,33 (1,14%)	1,6 ± 0,03 (303,14%)	0,67 ± 0,03 (159,10%)
*N* = 3 Between Groups *p* = 0,002 (ANOVA) Between Control and 10 ng/mL TNF-α Assay Groups *p* = 0,005 (Tukey’s HDS) Between 10 ng/mL TNF-α Assay and 10 ng/mL TNF-α + Conditioned Medium Assay Groups *p* = 0,002 (Tukey’s HDS) Between 10 ng/mL TNF-α Assay Group and Conditioned Medium Assay Groups *p* = 0,003 (Tukey’s HDS)				
KDR	−0,015 ± 0,065 (96,59%)	−5,3 ± 0,0345 (2,53%)	−2,3 ± 2,45 (20,30%)	0,95 ± 0,64 (193,18%)
*N* = 3 Between Groups *p* = 0,036 (ANOVA) Between 10 ng/mL TNF-α Assay Group and Conditioned Medium Assay Groups *p* = 0,033 (Tukey’s HDS)				
vWF	0,69 ± 0,28 (161,32%)	−2,71 ± 0,755 (15,28%)	1,17 ± 0,5 (225,01%)	1,66 ± 1,56 341,01%)
*N* = 3 Between Groups *p* = 0,027 (ANOVA) Between Control and 10 ng/mL TNF-α Assay Groups *p* = 0,011 (Tukey’s HDS) Between 10 ng/mL TNF-α Assay and 10 ng/mL TNF-α + Conditioned Medium Assay Groups *p* = 0,005 (Tukey’s HDS) Between 10 ng/mL TNF-α Assay Group and Conditioned Medium Assay Groups *p* = 0,005 (Tukey’s HDS)				
ASCL4	−1,02 ± 0,45 (49,31%)	−0,65 ± 0,08 (63,72%)	−0,92 ± 0,32 (52,85%)	−0,59 ± 0,49 (66,43%)
*N* = 3 Between Groups *p* > 0,05 (ANOVA)				
NRF2	−1,27 ± 0,62 (41,46%)	−3,39 ± 0,07 (9,53%)	−1,32 ± 0,65 (40,05%)	−1,65 ± 0,26 (31,86%)
*N* = 3 Between Groups *p* = 0,04 (ANOVA) Between Control and 10 ng/mL TNF-α Assay Groups *p* = 0,04 (Tukey’s HDS) Between 10 ng/mL TNF-α Assay and 10 ng/mL TNF-α + Conditioned Medium Assay Groups *p* = 0,04 (Tukey’s HDS)				

## Discussion

The present study provides evidence that sustained, membrane-restricted CXCL10 signaling may actively drive mesenchymal stem cells toward an immunoregulatory transcriptional phenotype, thereby enhancing their paracrine capacity to modulate inflammatory endothelial responses. While CXCL10 is classically recognized as an interferon-inducible inflammatory chemokine involved in leukocyte recruitment via CXCR3, our findings extend its functional relevance beyond chemotaxis, suggesting a role in shaping the intrinsic immunomodulatory programming of MSCs.

CXCL10 has been extensively studied in the context of immune cell trafficking and inflammatory amplification. Its interaction with CXCR3 is known to promote the migration and activation of T lymphocytes, monocytes, and natural killer cells at sites of tissue injury. In the context of MSC biology, CXCL10–CXCR3 signaling has primarily been associated with MSC homing to inflamed tissues, and impairment of this axis has been shown to limit MSC therapeutic efficacy. However, whether CXCL10 directly influences the immunoregulatory identity of MSCs has remained insufficiently explored. Our study addresses this gap by demonstrating that constitutive membrane anchoring of CXCL10 is associated with a coordinated upregulation of key immunomodulatory mediators, including IDO1, TGF-β1, and IL4I1—molecules critically involved in immune tolerance, T-cell suppression, and anti-inflammatory macrophage polarization.

A central conceptual advance of this work lies in the spatial presentation of CXCL10 at the MSC surface, which serves as an active signaling trigger rather than a mere membrane modification. By tethering CXCL10 to the cell membrane via the Lactadherin C1/C2 domain, we aimed to mimic immune synapse–like interactions and enable sustained, contact-dependent CXCL10–CXCR3 signaling. This spatial restriction contrasts with conventional soluble chemokine stimulation; our findings indicate that localized, membrane-tethered CXCL10 transcriptionally reprograms the immunosuppressive profile of the cell by triggering a coordinated JAK2/STAT and NF-κB signaling cascade. Indeed, the significant increase observed in IDO1 and TGF-β1 mRNA expression levels is supported by the robust topological connectivity identified in our in silico interactome analysis. Collectively, these results corroborate the hypothesis that surface-localized CXCL10 facilitates the transcriptional stabilization of the cell’s metabolic effector module (IDO1 and IL4I1) through a constitutive juxtacrine loop, biasing the MSCs toward a stable immunoregulatory phenotype.

Consistent with this interpretation, conditioned medium derived from CXCL10-LACTC1/C2 expressing MSCs attenuated TNF-α–induced endothelial activation, as reflected by the modulation of adhesion molecules and endothelial functional markers. Reduced ICAM-1 expression and restoration of PECAM-1 levels suggest a diminished endothelial inflammatory state and improved intercellular junction integrity. In parallel, the recovery of KDR expression and enhanced endothelial viability indicate that the MSC secretome may promote endothelial repair and survival under inflammatory stress. Together, these observations suggest that CXCL10-mediated reprogramming of MSCs enhances their capacity to regulate leukocyte–endothelial interactions and preserve vascular homeostasis.

Beyond classical inflammatory markers, our analysis also explored pathways related to oxidative stress and ferroptosis. Although TNF-α stimulation altered NRF2 expression, the presence of conditioned medium from CXCL10-modified MSCs partially restored NRF2 levels, suggesting a potential role in redox homeostasis. In contrast, ACSL4 expression remained largely unchanged, indicating that ferroptotic signaling may not be a dominant mechanism under the experimental conditions employed. These findings suggest that the protective effects of the MSC secretome are more closely linked to modulation of inflammatory and oxidative stress responses rather than direct regulation of lipid peroxidation pathways.

Taken together, our data support a model in which membrane-anchored CXCL10 functions not merely as a chemotactic cue, but as a spatially organized signaling module capable of reprogramming MSC immunomodulatory function. This reprogramming enhances the paracrine capacity of MSCs to counteract cytokine-driven endothelial dysfunction. From a broader perspective, these findings highlight the importance of chemokine spatial presentation in stromal cell biology and suggest that engineering sustained, contact-dependent chemokine signaling may represent a promising strategy to enhance MSC-based and cell-free therapeutic approaches for inflammation-driven vascular pathologies.

Although mechanistic insights were provided in the present study, several limitations should be acknowledged. First, the interaction between membrane-anchored CXCL10 and CXCR3 would benefit from further validation using high-resolution imaging approaches or reductionist signaling assays to directly demonstrate receptor engagement and downstream activation. Second, the functional consequences of CXCL10–CXCR3 signaling could be more definitively confirmed through the use of CXCR3-neutralizing antibodies, pharmacological inhibitors, or CXCR3-deficient MSC models.

Third, the findings of this study were primarily derived from basic in vitro models. As such, they may not fully recapitulate the entire spectrum of endothelial responses under inflammatory conditions or the complexity of the inflammatory vascular microenvironment; therefore, validation using in vivo systems or more advanced co-culture models will be important in future studies.

Finally, comprehensive proteomic profiling of the secretome derived from CXCL10–LACT-C1/C2 expressing MSCs would provide additional support and deeper insight into the molecular alterations underlying the observed immunomodulatory effects.

## Conclusion

It has been demonstrated that the secretome present in the conditioned medium derived from CXCL10-LACTC1/C2⁺ MSC cultures effectively counteracts the pro-inflammatory effects induced by TNF-α in HUVECs. This protective effect appears to be strongly associated with the mRNA expression of key immunoregulatory genes including IDO1, IL4I1, and TGF-β1 in CXCL10-LACTC1/C2⁺ MSCs. Furthermore, the data indicate that the underlying mechanism operates through CXCL10–CXCR3 signaling, potentially facilitated by cell-cell contact. These findings suggest that CXCL10–CXCR3 signaling not only directs the migration and homing of MSCs toward inflammatory microenvironments, as previously described, but may also promote the acquisition of anti-inflammatory properties in these cells.

## Supplementary Information

Below is the link to the electronic supplementary material.

Supplementary Material 1 (DOCX 17.7 KB)

Supplementary Material 2 (DOCX 14.5 KB)

Supplementary Material 3 (DOCX 15.2 KB)

Supplementary Material 4 (DOCX 24.6 KB)

Supplementary Material 5 (DOCX 14.1 KB)

Supplementary Material 6 (DOCX 20.5 KB)

Supplementary Material 7 (DOCX 736 KB)

## Data Availability

The datasets generated and/or analyzed during the current study are available from the corresponding author upon reasonable request.

## Declarations

### Use of Artificial Intelligence Tools

During the preparation of this manuscript, an artificial intelligence–based language assistance tool (ChatGPT, OpenAI) was used solely to improve English grammar, sentence structure, and overall clarity of the text. The tool was not used for study design, data generation, data analysis, interpretation of results, or formulation of scientific conclusions. All experimental procedures, data analyses, and interpretations were performed and validated by the authors, who take full responsibility for the content of the manuscript.
