# Supplementary material for: CXCL10-LACTC1/C2 Expressing Mesenchymal Stem Cell Conditioned Medium Attenuates TNF-α-Induced Gene Expressions and Cell Viability in HUVECs
Source: Inflammation. 2026 May 22;49(1):164. doi: 10.1007/s10753-026-02518-2 (PMC13369754; doi:10.1007/s10753-026-02518-2)
Supplement: Supplementary file 1 — Supplementary Material 1 (DOCX 17.7 KB) [file 10753_2026_2518_MOESM1_ESM.docx]

**Supplementary Material 1:** We have validated the functionality and infectivity of the manufactured lentiviruses through a titration assay prior to the main experiments.

To determine the optimal dosage, hWJ-MSCs were transduced with varying volumes of the concentrated pseudovirus suspension (50, 100, and 200 µL). As shown in **Supplementary Material 6** Lentivirus Titration, we observed a dose-dependent increase in GFP fluorescence intensity. Based on these results, **200 µL of virus suspension** was identified as the optimal dosage, providing the highest transduction efficiency without compromising cell viability.
