# Supplementary material for: CXCL10-LACTC1/C2 Expressing Mesenchymal Stem Cell Conditioned Medium Attenuates TNF-α-Induced Gene Expressions and Cell Viability in HUVECs
Source: Inflammation. 2026 May 22;49(1):164. doi: 10.1007/s10753-026-02518-2 (PMC13369754; doi:10.1007/s10753-026-02518-2)
Supplement: Supplementary file 2 — Supplementary Material 2 (DOCX 14.5 KB) [file 10753_2026_2518_MOESM2_ESM.docx]

Supplementary Material 2. HUVEC culture groups, composition of culture media, and incubation periods

| Group Name | Medium Composition | Total Incubation Time |
| --- | --- | --- |
| Control HUVEC Cultures | Medium 200 containing Large Vessel Endothelial Supplement (LVES). | 72 hours (medium was refreshed every 24 hours). |
| Experimental HUVEC Cultures | Medium 200 containing 10 ng/mL TNF-alpha and LVES. | HUVEC cultures were conditioned for 24 hours. |
| TNF-alpha Experimental Group | Medium 200 containing 10 ng/mL TNF-alpha and LVES. | 48 hours (medium was refreshed every 24 hours). |
| TNF-alpha and CXCL10-LACTC1/C2 MSC Conditioned Medium Experimental Group | Medium 200 containing 10 ng/mL TNF-alpha, 30% (v:v) CXCL10-LACTC1/C2 MSC conditioned medium, and LVES. | 48 hours (medium was refreshed every 24 hours). |
| CXCL10-LACTC1/C2 MSC Conditioned Medium Experimental Group | Medium 200 containing 30% (v:v) CXCL10-LACTC1/C2 MSC conditioned medium and LVES. | 48 hours (medium was refreshed every 24 hours). |

*Note: All control and experimental groups underwent a total incubation period of 72 hours. Experimental groups were initially conditioned with 10 ng/mL TNF-alpha for 24 hours, after which they were incubated in the respective media described in the table for an additional 48 hours.*
