# Supplementary material for: CXCL10-LACTC1/C2 Expressing Mesenchymal Stem Cell Conditioned Medium Attenuates TNF-α-Induced Gene Expressions and Cell Viability in HUVECs
Source: Inflammation. 2026 May 22;49(1):164. doi: 10.1007/s10753-026-02518-2 (PMC13369754; doi:10.1007/s10753-026-02518-2)
Supplement: Supplementary file 3 — Supplementary Material 3 (DOCX 15.2 KB) [file 10753_2026_2518_MOESM3_ESM.docx]

**Supplementary Material 3. Forward and Reverse Primers for Real Time Polymerase Chain Reactions**

| Gene | Forward Primer 5’-3’ | Reverse Primer 5’-3’ | Details |
| --- | --- | --- | --- |
| Beta 2 Mikroglobulin | CTGGCGGGCATTCCTGA | TGGAGTACGCTGGATAGCCT | House keeping gene |
| CXCL10-LACTC1/C2 | TGAATCCAGAAAATCGAAGGCCA | GCGATCTGTGAGTTGGCAAT | For modified MSCs |
| CXCR3 | GTGACTCGTGCTGTAACCTCC | TAGGTGGAGCAGGAAGGTGT | For modified MSCs |
| IL4I1 | CCATCAAGATCAACAGCCGG | TAACTGGCCTTGGACTGGAG | For modified MSCs |
| IDO1 | GCAAATGCAAGAACGGGACAC | GTGTGAAAGCTCTGGTCTCCCTGA | For modified and control MSCs |
| TGF-β1 | ACCTGCCACAGATCCCCTAT | CGGTAGTGAACCCGTTGATG | For modified and control MSCs |
| ICAM-1 | CCTACCAGCTCCAGACCTTT | AAGGAGTCGTTGCCATAGGT | Control and assay groups of HUVECs |
| PECAM-1 | AAGCAGAAGGCTAGCAAGGA | CAGCTTTCCGGACTTCACTG | Control and assay groups of HUVECs |
| KDR | CGGTCAACAAAGTCGGGAGA | CAGTGCACCACAAAGACACG | Control and assay groups of HUVECs |
| vWF | GGTGGCTCGACATTTAGG | GGTGGAGGACTTCGGGAAC | Control and assay groups of HUVECs |
| ACSL4 | GGAATGACAGGCCAGTGTGA | TCCGGAACAGCAGCCATAAG | Control and assay groups of HUVECs |
| NRF2 | GAGCAAGTTTGGGAGGAGCT | GGTTGGGGTCTTCTGTGGAG | Control and assay groups of HUVECs |
