# Supplementary material for: CXCL10-LACTC1/C2 Expressing Mesenchymal Stem Cell Conditioned Medium Attenuates TNF-α-Induced Gene Expressions and Cell Viability in HUVECs
Source: Inflammation. 2026 May 22;49(1):164. doi: 10.1007/s10753-026-02518-2 (PMC13369754; doi:10.1007/s10753-026-02518-2)
Supplement: Supplementary file 4 — Supplementary Material 4 (DOCX 24.6 KB) [file 10753_2026_2518_MOESM4_ESM.docx]

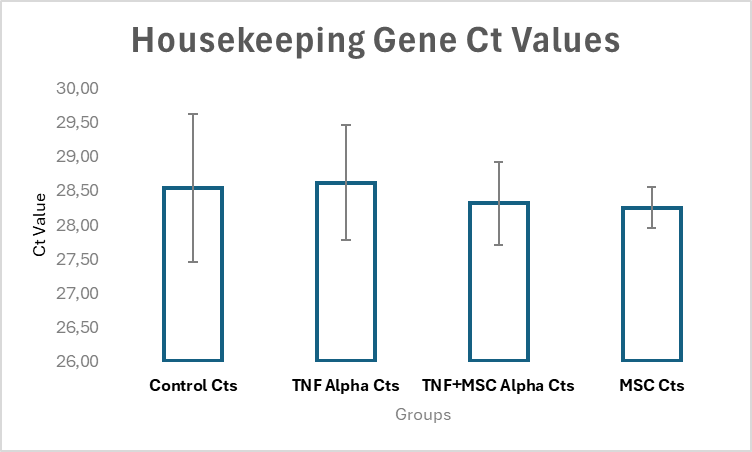


| HUVECs [Control Group Mean Ct] | HUVECs treated with TNF-α alone [TNF Alpha Mean Ct] | HUVECs treated with TNF-α and CXCL10-LACTC1/C2 MSCs^+^ CM [TNF Alpha +MSC Mean Ct] | HUVECs treated with CXCL10-LACTC1/C2 MSCs^+^ CM alone [MSC Mean Ct] |
| --- | --- | --- | --- |
| 28.55±1.09 | 28.62±0,84 | 28.32±0,61 | 28.25±0,30 |

**Supplementary Material 4 :** Housekeeping Gene Stability, there were no significant differences in B2M expression levels between the inflammatory (TNF-alpha treated) and non-inflammatory groups (**p = 0.83**, One-way ANOVA).
