# Supplementary material for: CXCL10-LACTC1/C2 Expressing Mesenchymal Stem Cell Conditioned Medium Attenuates TNF-α-Induced Gene Expressions and Cell Viability in HUVECs
Source: Inflammation. 2026 May 22;49(1):164. doi: 10.1007/s10753-026-02518-2 (PMC13369754; doi:10.1007/s10753-026-02518-2)
Supplement: Supplementary file 5 — Supplementary Material 5 (DOCX 14.1 KB) [file 10753_2026_2518_MOESM5_ESM.docx]

**Supplementary Material 5. Antibodies for IFA**

| **Antibody** | **Localization** | **Cat no** | **Host** | **Dilution** |
| --- | --- | --- | --- | --- |
| CXCL10 | Membrane | ThermoFisher Scientific / 10H11L3 | Rabbit | 1:150 |
| Vimentin | Cytoplasmic | Invitrogen / MA5-11883 | Mouse | 1:150 |
| CD44 | Membrane | Invitrogen / MS-668-P | Mouse | 1:150 |
| Goat anti-mouse | FITC conjugate | Invitrogen / 31569 | Goat | 1:200 |
| Goat anti-mouse | AF-647 conjugate | CST / 4110S | Goat | 1:200 |
