# Supplementary material for: CXCL10-LACTC1/C2 Expressing Mesenchymal Stem Cell Conditioned Medium Attenuates TNF-α-Induced Gene Expressions and Cell Viability in HUVECs
Source: Inflammation. 2026 May 22;49(1):164. doi: 10.1007/s10753-026-02518-2 (PMC13369754; doi:10.1007/s10753-026-02518-2)
Supplement: Supplementary file 6 — Supplementary Material 6 (DOCX 20.5 KB) [file 10753_2026_2518_MOESM6_ESM.docx]

**Supplementary Material 6. Materials**

| **Material** | **Supplier** | **Catalog No** |
| --- | --- | --- |
| *Cells* | | |
| Human Wharton’s Jelly Mesenchymal Stem Cells | Tissuecare Biotechnology | A01- hWJ-MSCs |
| Human umbilical vein endothelial cells (HUVEC) | Thermo Fisher | C0035C |
| *Cell Culture Reagents* | | |
| DMEM/F12 | Thermo Fisher | 11320033 |
| DPBS | Thermo Fisher | 14190144 |
| Fetal bovine serum (FBS) | Thermo Fisher | 12662029 |
| L-gltamine | Thermo Fisher | 25030081 |
| Penicillin-streptomycin | Thermo Fisher | 15140-122 |
| Trypsin-EDTA | Thermo Fisher | 25300054 |
| Medium200 | Thermo Fisher | M200500 |
| Large Vessel Endothelial Supplement (LVES) | Thermo Fisher | A1460801 |
| Human TNF-Alpha Recombinant Protein | Thermo Fisher | PHC3011 |
| Cells and Reagents for Lentivirus Production | | |
| LentiSuit Deluxe Kit | System Biosciences | LV350A |
| *Plasmids* | | |
| pLENTI-III-EF1a-MEFGE8-Vector | Applied Biological Materials | Modified as requested. |
| Flow Cytometry Antibodies | | |
| Human Mesenchymal Stem Cell Verification Multi-Color Flow Cytometry Kit | R&D Systems | FMC020 |
| *RNA Isolation, Reverse PCR and qPCR Reagents* | | |
| Total RNA Isolation Kit | Ecopure | E2075 |
| High Capacity cDNA Reverse Transcription Kit | Applied Biosystems | 4368814 |
| PowerUp™ SYBR™ Green Master Mix | Applied Biosystems | A25742 |
| *SDS PAGE and Western Blotting Reagents* | | |
| RIPA Lysis Buffer System, | Santa Cruz Biotechnology | sc-24948, |
| Quick Start Bradford Protein Assay | Bio-rad | 5000202 |
| Ammonium Persulfate (APS) | Sigma Aldrich | A3678 |
| Sodium Dodecyl Sulfate (SDS) | Sigma Aldrich | L3771 |
| Tris base | Sigma Aldrich | 93362 |
| Glycine | Sigma Aldrich | G7126 |
| Page Ruler Plus PreStained Ladder (10–250 kDa) | Thermo Fisher | 26619 |
| 2X Laemmli buffer | Bio-rad | 1610737 |
| 0.2 μm PVDF mini membranes (Biorad, Transfer Pack) | Bio-rad | 1704156 |
| CXCL10 primary antibody | Thermo Fisher | 10H11L3 |
| Goat Anti-Rabbit IgG secondary antibody HRP Conjugated | Abcam | ab97051 |
| Excellent Chemiluminancent Substrate (ECL) | Elabscience | E-IR-R301 |
| *Immunofluorescence Staining* | | |
| CXCL10 | Thermo Fisher | 10H11L3 |
| Vimentin | Invitrogen | MA5-11883 |
| CD44 | Invitrogen | MS-668-P |
| Goat anti-mouse-FITC conjugated | Invitrogen | 31569 |
| Goat anti-mouse-AF-647 conjugated | CTS | 4110S |
| Triton X | Sigma Aldrich | T8787 |
| Bovine Serum Albumin (BSA) | Sigma Aldrich | B6917 |
| Viability Testing | | |
| MTT Powder | Sigma Aldrich | M2128 |
| Phosphate-buffered saline (PBS) | Thermo Fisher | P4474 |
