# Supplementary figures and images for: CXCL10-LACTC1/C2 Expressing Mesenchymal Stem Cell Conditioned Medium Attenuates TNF-α-Induced Gene Expressions and Cell Viability in HUVECs
Source: Inflammation. 2026 May 22;49(1):164. doi: 10.1007/s10753-026-02518-2 (PMC13369754; doi:10.1007/s10753-026-02518-2)

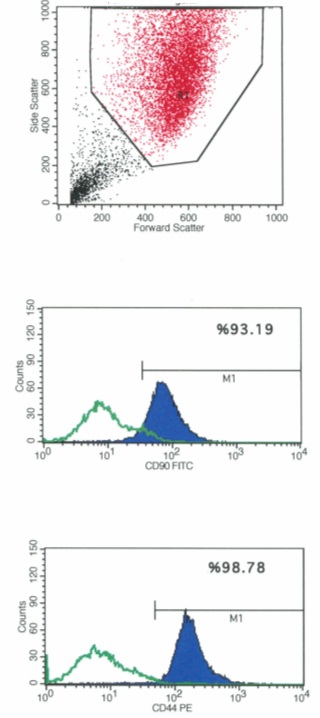


**Supplementary Materil 7:** Flow Cytometric Immunophenotyping of hWJ-MSCs

Supplement: Supplementary file 7 — Supplementary Material 7 (DOCX 736 KB) [file 10753_2026_2518_MOESM7_ESM.docx]
